# Editorial: NKT Cells in Cancer Immunotherapy

**DOI:** 10.3389/fimmu.2020.01314

**Published:** 2020-06-23

**Authors:** Tonya J. Webb, Weiming Yuan, Everett Meyer, Paolo Dellabona

**Affiliations:** ^1^Department of Microbiology and Immunology, Greenebaum Comprehensive Cancer Center, University of Maryland School of Medicine, Baltimore, MD, United States; ^2^Department of Molecular Microbiology and Immunology, Norris Comprehensive Cancer Center, Keck School of Medicine, University of Southern California, Los Angeles, CA, United States; ^3^Division of Blood and Marrow Transplantation, Stanford University School of Medicine, Stanford, CA, United States; ^4^Experimental Immunology Unit, Division of Immunology, Transplantation and Infectious Diseases, IRCCS San Raffaele Scientific Institute, Milan, Italy

**Keywords:** iNKT, CD1d, dendritic cells, α-GalCer, cancer, immunotherapy

Natural killer T (NKT) cells are a unique subset of T cells that recognize glycolipid antigens within the context of CD1d, a non-classical MHC class I-like molecule (1–3). NKT cells have the capacity to mount strong anti-tumor responses and have thus become a major focus in the development of effective cancer immunotherapy. Type I, invariant NKT (iNKT) cells, are the most well-characterized subset of CD1d-restricted T cells. NKT cells express an invariant Vαβ TCR and are known for their ability to rapidly produce copious amounts of Th1, Th2, and Th17-type cytokines following stimulation by CD1d-antigen complexes (4). α-Galactocylceramide (α-GalCer) is a potent activator of iNKT cells. Following treatment with α-GalCer, iNKT cells produce large amounts of cytokines, undergo clonal expansion, and subsequently activate NK cells, neutrophils, macrophages, dendritic cells (DC), B cells, and T cells. Moreover, activated NKT cells can directly induce cell death in tumor cells and infected cells. NKT cells have been shown to play a critical role autoimmune disease, infection, transplant immunology, and cancer. Therefore, it is important to understand how to effectively guide their effector functions in order to develop novel immunotherapeutic strategies (Lam et al.). The articles in this special issue are centered around our current understanding of NKT cell biology and address outstanding questions in the field.

α-GalCer has been utilized extensively due to its ability to induce potent activation of mouse and human iNKT cells. In this collection, Zhang et al. highlight different modalities for mobilizing iNKT cells for anti-cancer therapies. These studies are important because preclinical studies have shown that repeated exposure to α-GalCer can result in iNKT cell anergy. It is now appreciated that loading dendritic cells with glycolipid antigens can help avoid the induction of anergy. Fujii and Shimizu focused on NKT-mediated immunotherapy through selective DC targeting. Other approaches include using nanovectors/nanoparticle-based delivery systems, α-GalCer loaded exosomes, as well as treatment with IL-2 or using antibodies to block inhibitory signaling (Ghinnagow et al.; Lam et al.; Zhang et al.). Co-signaling molecules, such as CD28 and PD-1, can positively and negatively influence iNKT cell activation, and a review from the Webb lab details the costimulatory requirements for iNKT cell development and function (Shissler et al.).

New approaches to improve preclinical platforms to study α-GalCer-based immunotherapies include the development of humanized CD1d/NKT mouse models by several groups (Zhang et al.). In addition, Dashtsoodo et al. has developed a therapy using a newly identified NKT cell agonist, RK. They found that RK-pulsed DCs resulted in the establishment of long-term T cell memory responses. Strikingly, treatment of B16-melanoma bearing mice with RK-pulsed DCs, resulted in a nearly complete elimination of the tumor, whereas treatment with a similar concentration of α-GalCer-pulsed DCs did not demonstrate any therapeutic benefit.

Studies by Wolf et al., discussed clinical trials focused on activating iNKT cells using α-GalCer pulsed antigen presenting cells, as well as adoptive iNKT cell therapies using blood-derived *ex vivo* expanded iNKT cells. This and other related work suggests that iNKT-CARs may be advantageous because their endogenous TCR has intrinsic anti-tumor activity, strong signaling through the TCR typically results in a Th1-type cytokine bias in NKT cells, and iNKT cells can migrate into non-lymphoid tissues; therefore, they could mediate anti-tumor immune responses in non-lymphoid tumors. Aside from the fact that iNKT-CAR could be developed more easily as a third-party cellular therapy, iNKT may behave differently than conventional CAR T for which exhaustion and anergy limit their efficacy in non-lymphoid tumors. In this vein, Zhang and Donda has developed bi-functional fusion proteins composed of extracellular CD1d and antibody scFv fragments specific to HER2 or CEA as a means of redirecting iNKT cells to the tumor site. Importantly, they found that treatment of tumor-bearing mice with their α-GalCer-loaded-CD1d anti-tumor fusion proteins resulted in the recruitment of iNKT, NK, and T cells to the tumor, leading to a significant reduction in tumor growth.

Mavers et al. highlights the critical role of NKT cells in reducing graft vs.-host-disease (GVHD) in preclinical and clinical studies of allogeneic hematopoietic stem cell transplantation and enhancing anti-tumor immune reactions (GVT). Their work also highlights two key areas needed to advance iNKT into clinical practice, including the need to better define and recapitulate NKT cell subsets and a better understanding of optimal drug delivery strategies for α-GalCer or other glycolipids for the activation and modulation of the appropriate NKT cell subset *in vivo*. It has been well-established that NKT cells can be activated following their recognition of lipid antigen presented in the context of CD1d or by cytokines. Modes of NKT cell activation are discussed by Cerundolo's lab as a means to understanding how to effectively harness their effector functions in cancer immunotherapy (Bedard et al.). Advances in technology, such as single cell RNA-seq and microfluidics, can help to provide a detailed description of the specific NKT subsets within the tumor microenvironment. Teyton et al. discusses how the implementation of these techniques can be used to gain a better understanding of NKT cell during tumorigenesis.

Type II NKT cells have been shown to play a suppressive role in many different disease settings; however, due to limitations in reagents and model systems it has been difficult to study this unique CD1d-restricted subpopulation. Herein, Kato et al. discuss experimental tools that can be used to analyze type II NKT cells, such as, 24αβ-TCR mice, 4get Jα18 ^−/−^ mice and CD1d tetramers. The review by Nair and Dhadapkar also discusses suppression of tumor immunity by type II NKT cells. Importantly, this comprehensive issue includes primary research investigating the impact of neurofibromin 1 on CD1d expression (Liu et al.) and discusses NKT cells from an ecological, evolutionary, and developmental biology “eco-evo-devo” perspective (Kumar et al.). The gut microbiota has been demonstrated to play a critical role determining responses to immune checkpoint inhibitor therapy, and NKT cells have been shown to regulate gut microbial ecology, thus future studies should investigate the impact of the gut microbiome and NKT cell number and function on immune responses and patient outcomes following treatment with cancer immunotherapy.

This special issue also describes models in which type I NKT cells have been shown to play a suppressive role in Th1 responses, thereby promoting tumor formation, specifically in the intestines (Wang and Cardell). Given the dual role that NKT cell can play in anti-tumor immunity, by either inducing tumor suppression or promoting tumor formation, it is important to investigate the role of the local tumor microenvironment and understand how this guides NKT cell responses (5, 6). Also, it is critical to identify and specific markers, such as PLZF, that can help define functional differences within particular NKT cell subsets. In closing, this special issue is a collection of reviews and research articles that showcases the pioneering techniques, unique model systems, and innovative therapeutic strategies being utilized to modulate NKT cells. This work in combination with future studies, will aid our understanding of how to effectively manipulate these potent effector cells in order to induce optimal anti-tumor immune responses.

## Author Contributions

All authors listed have made a substantial, direct and intellectual contribution to the work, and approved it for publication.

## Conflict of Interest

TW was the CEO of WebbCures, LLC, co-founded Screen Therapeutics and serves as a scientific advisor for Immunaccel. The remaining authors declare that the research was conducted in the absence of any commercial or financial relationships that could be construed as a potential conflict of interest.
