# Erratum: Editorial: NKT Cells in Cancer Immunotherapy

**DOI:** 10.3389/fimmu.2020.594504

**Published:** 2020-08-31

**Authors:** Frontiers Production Office

**Affiliations:** Frontiers Media SA, Lausanne, Switzerland

**Keywords:** iNKT, CD1d, dendritic cells, α-GalCer, cancer, immunotherapy

Due to a production error, reference 5 was incorrectly written as “Shissler SC, Webb TJ. The ins and outs of type I iNKT cell development. *Mol Immunol*. (2019) 105:116–30. doi: 10.1016/j.molimm.2018.09.023.” It should be “Shissler SC, Lee MS, Webb TJ. Mixed signals: co-stimulation in invariant natural killer T cell-mediated cancer immunotherapy. *Front Immunol*. (2017) 8:1447. doi: 10.3389/fimmu.2017.01447”.

 The publisher apologizes for this mistake. The original article has been updated.

